# Cellular therapy of corneal epithelial defect by adipose mesenchymal stem cell-derived epithelial progenitors

**DOI:** 10.1186/s13287-019-1533-1

**Published:** 2020-01-03

**Authors:** Francisco Bandeira, Tze-Wei Goh, Melina Setiawan, Gary Hin-Fai Yam, Jodhbir S. Mehta

**Affiliations:** 1 Tissue Engineering and Stem Cell Group, Singapore Eye Research Institute, 20 College Road, The Academia, Discovery Tower Level 6, Singapore, 169856 Singapore; 2 Federal University of São Paulo, Sao Paulo, Brazil; 3 Eye-Academic Clinical Program, Duke-National University of Singapore (NUS) Graduate Medical School, Singapore, Singapore; 4 Singapore National Eye Centre, Singapore, Singapore; 5 School of Material Science and Engineering, Nanyang Technological University, Singapore, Singapore

**Keywords:** Corneal epithelium, Limbal stem cell deficiency, Adipose mesenchymal stem cells, Mesenchymal-epithelial transition, Epithelial reconstruction

## Abstract

**Background:**

Persistent epithelial defects (PED), associated with limbal stem cell deficiency (LSCD), require ocular surface reconstruction with a stable corneal epithelium (CE). This study investigated CE reformation using human adipose mesenchymal stem cells (ADSC), which derived epithelial progenitors via mesenchymal-epithelial transition (MET).

**Methods:**

STEMPRO human ADSC were cultured with specific inhibitors antagonizing glycogen synthase kinase-3 and transforming growth factor-β signaling, followed by culture under a defined progenitor cell targeted-epithelial differentiation condition to generate epithelial-like cells (MET-Epi), which were characterized for cell viability, mesenchymal, and epithelial phenotypes using immunofluorescence and flow cytometry. Tissue-engineered (TE) MET-Epi cells on fibrin gel were transplanted to corneal surface of the rat LSCD model caused by alkali injury. Epithelial healing, corneal edema, and haze grading, CE formation were assessed by fluorescein staining, slit lamp bio-microscopy, anterior segment optical coherence tomography, and immunohistochemistry.

**Results:**

CD73^high^/CD90^high^/CD105^high^/CD166^high^/CD14^negative^/CD31^negative^ human ADSC underwent MET, giving viable epithelial-like progenitors expressing δNp63, CDH1 (E-cadherin), epidermal growth factor receptor, integrin-β4, and cytokeratin (CK)-5, 9. Under defined epithelial differentiation culture, these progenitors generated MET-Epi cells expressing cell junction proteins ZO1 and occludin. When transplanted onto rat corneal surface with LSCD-induced PED, TE-MET-Epi achieved more efficient epithelial healing, suppressed corneal edema, and opacities, when compared to corneas without treatment or transplanted with TE-ADSC. CE markers (CK3, 12, and CDH1) were expressed on TE-MET-Epi-transplanted corneas but not in other control groups.

**Conclusion:**

Human ADSC-derived epithelial-like cells, via MET, recovered the CE from PED associated with LSCD. ADSC can be a viable adult stem cell source for potential autologous epithelial cell-based therapy for corneal surface disorders.

## Background

The corneal epithelium (CE) is the outermost layer of the cornea. It is about 50 μm thick and consists of 5–7 layers of the stratified squamous non-keratinized epithelium [1]. The basal layer contains cuboidal cells adhered to an underlying basement membrane, the Bowman’s membrane. The CE is a self-renewing tissue, owing to the epithelial stem cells (limbal stem cells, LSC) present in the limbal basal epithelium at the corneal periphery [2, 3]. This unique population of epithelial progenitors constantly provides new cells for normal epithelial turnover and wound healing. The transparent CE maintains corneal clarity and protects the eye against infection and damage while allowing nutrient transfer and gaseous exchange from tear fluid. A breach of CE integrity, due to mechanical trauma (like foreign body intrusion, contact lens overuse, and chemical burns); infection, neurotrophic keratopathy, dry eye, systemic and genetic disorders (e.g., thyroid eye diseases, Sjogren’s syndrome, aniridia-related keratopathy caused by *Pax6* mutations, and ectodermal dysplasia caused by *P63* mutations), and limbal stem cell deficiency (LSCD); causes persistent epithelial defects (PED), which result in corneal scarring, ulceration, neovascularization, conjunctivalization and, ultimately, corneal opacification, and visual loss [4].

The management of severe CE defects is challenging. When medical treatments fail and the defects or ulcer persist (for more than 3 weeks), conventional surgical treatments become indicated [5]. In severe cases, the disorders could have destroyed LSC population and compromised its regenerative capacity, resulting in LSCD. In bilateral total LSCD, there are no autologous cell sources to reconstruct the damaged ocular surface. Corneal grafting in these conditions is frequently indicated and demands a replacement of healthy corneolimbal epithelium, with stem cell population (keratolimbal grafting) from donor corneas [6]. Though it has shown significance in improving the visual acuity in patients with bilateral LSCD, allograft rejection remains the most common cause of long-term epithelial failure. Patients usually require a prolonged course of systemic immunosuppression, which could cause adverse effects, including hyperglycemia, elevated creatinine, and hypertension, as well as elevated intraocular pressure and cataract [7, 8]. Adult tissue-specific MSC (mesenchymal stem cells) have been introduced as an accessible and non-immunogenic stem cell source, with potential therapeutic value in CE regeneration and treatment of PED for corneal surface disorders [9, 10]. These multipotent cells have the capacity to differentiate towards adipocyte, chondrocyte, and osteoblasts [11, 12]. Human adipose-derived MSC (ADSC) incubated in culture media conditioned with human CE cells attained polygonal morphology and upregulated transforming growth factor-β (TGFβ) receptor (CD105) and cytokeratin (CK)-12 (CE marker) [13]. Rabbit bone marrow MSC co-cultured with LSC displayed CK3 expression [14]. Although there have been promising results of significant CE regeneration, healing of PED and vision recovery in animal models, and clinical trial, it remains uncertain whether MSC can transdifferentiate into CE cells [15, 16]. Other actions include the secretion of trophic factors and cytokines to stimulate the surviving resident cells to proliferate and to exert anti-inflammatory and immunomodulatory effects on the injured corneal tissue [17, 18]. Our group has reported the mesenchymal-epithelial transition (MET) of human ADSC into epithelial lineage via antagonizing GSK3 (glycogen synthase kinase 3) and TGFβ signaling [19]. It generated epithelial-like progenitors expressing E-cadherin (CDH1), cytokeratins, epithelial proliferation markers (δNp63 and proliferating cell nuclear antigen) with concomitant suppression of N-cadherin (CDH2), indicating MET progression.

In this study, we examined the therapeutic potential of these ADSC-derived epithelial progenitors on CE reconstruction in a rat alkali-burn induced total LSCD model. Cells grown on thin fibrin gel and differentiated to form tissue-engineered (TE) epithelial construct were transplanted to an injured corneal surface. The effect on corneal epithelial healing, opacity, and edema, as well as CE marker expression, was examined and compared to injured control without treatment or transplanted with ADSC on fibrin gel.

## Methods

### Human primary ADSC culture and characterization

Human ADSC (*n* = 3) from StemPro Human Adipose-Derived Stem Cell Kit (ThermoFisher, Waltham, MA, USA) were cultured at 10^4^ cells/cm^2^ in MesenPRO RS™ medium (ThermoFisher) with 2% serum (provided in MesenPRO kit). Cells were subpassaged using TryPLE-Express (ThermoFisher). The multilineage differentiation potential was assessed by induced differentiation to adipocytes using StemPro Adipogenesis kit (ThermoFisher) to chondrocytes using the StemPro Chondrogenesis kit (ThermoFisher). The cells were stained with Oil Red O (Sigma-Aldrich, St. Louis, MI, USA) for adipocytes, toluidine blue (Sigma-Aldrich) for chondrocytes, and alizarin red S (Sigma-Aldrich) for osteocytes [19].

### Mesenchymal-epithelial transition

**Fig. 1:**
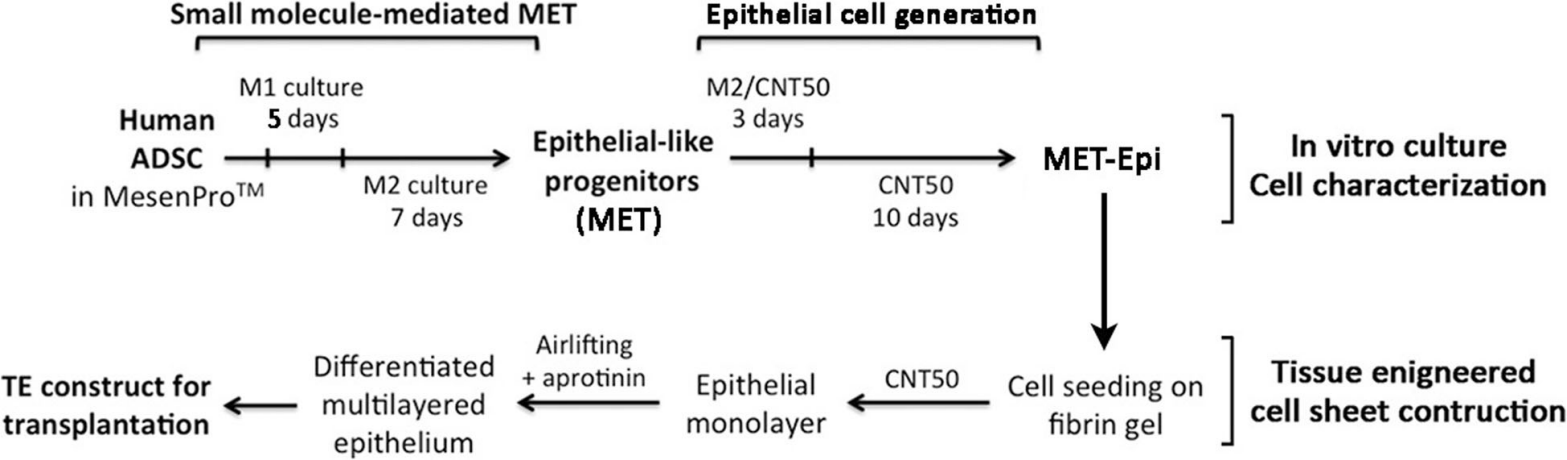
A schematic diagram showing the small molecule-induced conversion of human ADSC to epithelial-like progenitors via mesenchymal-epithelial transition (MET) and further differentiation to generate epithelial cells (MET-Epi). The cell sheet was tissue-engineered on fibrin gel to prepare construct for transplantation

Human ADSC at passage 2 were induced to undergo a mesenchymal-epithelial transition (MET) as previously reported [19] with modifications. A schematic diagram is depicted in Fig. 1. In brief, ADSC were placed in MET induction medium (M1) (Table 1) for 5 days. The culture was then changed to M2 medium with reduced VPA M and CHIR99021 concentration while other components were unchanged (Table 1). After M2 culture for 7 days, the cells were placed in M2/CNT-50 medium (CELLnTEC, Bern, Switzerland) (1:1 v/v) for 3 days, followed by CNT-50 for 10 days. Fresh medium was replenished every 3 days.

**Table 1:** Media formulation

Media	Basal media	Supplements
MET induction medium (M1)	MesenPRO-RS™ (ThermoFisher)	Vaporic acid (VPA, 500 μM; Sigma-Aldrich), CHIR99021 (3 μM; Stemgent), E-616452 (RepSox, 1 μM; Millipore), Tranylcypromine (5 μM; Tocris), A-83-01 (500 nM; Tocris) All-trans retinoic acid (atRA, 10 μM; Sigma-Aldrich) Serum (2%, supplied from MesenPro kit) Antibiotics-antimycotic (Invitrogen)
MET induction medium (M2)	MesenPRO-RS™	Same as M1 with reduced VPA (50 μM) and CHIR99021 (300 nM)
M2/CNT-50 medium		M2 and CNT-50 medium (CELLnTEC, Bern, Switzerland) (1:1 v/v)

### Tissue-engineered cells on fibrin gel construct

Plasminogen-free fibrinogen powder (Baxter, Singapore) was reconstituted in saline at a concentration of 5.3 mg/ml. Thrombin powder (Baxter) was dissolved in 0.9 mM calcium chloride at a concentration of 4 IU/ml. Fibrin gel (300 μl volume) was prepared by mixing fibrinogen with thrombin at 0.024 IU/ml and casted on culture insert at 37 **°**C for an hour for polymerization. Prior to cell seeding, the fibrin gel was rinsed with CNT-50 thrice to remove any unpolymerized molecules. ADSC-derived MET cells were seeded on fibrin gel surface at 5 × 10^4^ cells/cm^2^ in CNT50 medium (Fig. 1). Upon confluence, the cell/fibrin gel construct was airlifted by lowering the level of culture medium (CNT50 added with 0.5 IU/ml aprotinin) to submerge at the base of the culture insert. The construct was kept moist with sterile saline. After 7 days of stratification to cell multilayering, the construct was collected for transplantation. In parallel, human ADSC (without MET) were cultured on fibrin gel and underwent airlifting to provide a control construct for animal experiment. For human cell tracking, the epithelial construct was incubated with Molday ION Evergreen™ reagent (1:200 dilution; BioPAL, Worcester, MA, USA) for 48 h before surgery.

### Flow cytometry

Cell suspension after TryPLE digestion from culture was fixed with neutral-buffered 2% paraformaldehyde and blocked with 2% bovine serum albumin (BSA) and 2% normal goat serum (NGS, Sigma-Aldrich). The samples were incubated with antibodies against cell surface epitopes (CD14, 31, 73, 90, 105, 166, EGFR, ITGB4, OCLN and ZO1 (Additional file 1: Table S1), followed by AlexFluor488-conjugated IgG (Invitrogen). For the detection of intracellular epitopes (CDH1 and CDH2) (Additional file 1: Table S1), the cell samples were permeabilized with 1% Triton X-100 before blocking and antibody incubation. The staining signal was analyzed by FACSVerse System (BD Biosciences) with a minimum of 10,000 events in each experiment.

### Immunofluorescence

Cells grown on chamber slides (Lab-Tek II, Nunc) were fixed with neutral-buffered 2% paraformaldehyde, saponin-permeabilized, blocked with 2% BSA and NGS and incubated with antibodies (Additional file 1: Table S1) for 2 h at room temperature. After buffer washes, the samples were stained with AlexaFluor488 or 594-conjugated IgG antibody (Jackson ImmunoRes lab, West Grove, PA, USA), followed by mounting with Fluoroshield with DAPI (4′,6-diamidino-2-phenylindole; Santa Cruz Biotech., Santa Cruz, CA, USA) and viewed under fluorescence microscopy (Axioplan 2, Carl Zeiss, Oberkochen, Germany).

### Cell viability

The cell culture was incubated with MTT (3–4,5-dimethylthiazol-2-yl-2,5-diphenyltetrazolium bromide, 0.5 mg/ml) for 4 h, followed by addition of dimethylsulfoxide (DMSO, Sigma-Aldrich) to a final concentration of 5% to dissolve the formazan crystals. After centrifugation at 350 g for 5 min, the supernatant was collected and the optical density was measured at 560 nm (excitation) with a reference filter of 620 nm using a microplate reader (INFINITE 200, Tecan, Mannedorf, Switzerland). The relative cell number was calculated as the ratio of the optical density of cells under M1 or M2 culture to that of cells in MesenPRO™ culture. Experiments were done in triplicate.

### Rat LSCD model after alkali injury

Sprague-Dawley rats (4–8 weeks old, *n* = 24) were treated according to the regulation of the ARVO statement for the Use of Animals in Ophthalmic and Vision Research. The experimental protocol was approved by The Institutional Animal Care and Use Committee of SingHealth, Singapore (2012/SHS/768). Rats were anesthetized by intraperitoneal xylazine (5 mg/kg; Troy Laboratories, Glendenning, NSW, Australia) and ketamine hydrochloride (50 mg/kg; Parnell Laboratories, Alexandria, NSW, Australia). The eyes were rinsed with normal saline and anesthetized with topical lidocaine hydrochloride (1%; Pfizer Laboratories, NY, USA). Alkali injury was performed on the right eyes, after sterilization with povidone-iodine (Prodine, ICM Pharma, Singapore). A circular filter paper (4 mm inner diameter and 6 mm diameter) soaked in 0.5 N sodium hydroxide solution was placed on the corneal surface for 30 s, which was then washed with normal saline for 1 min with a minimum of 10 ml normal saline. CE was completely removed by gentle scraping with a #64 surgical blade. A 360° limbal peritomy was conducted to create total LSCD. After rinsing with normal saline, the corneal surface was ready for transplantation.

### Transplantation of TE cell/fibrin gel constructs

The rats were randomly placed in four groups for transplantation. Group 1: TE-MET-Epi/fibrin gel construct; group 2: TE-ADSC/fibrin gel construct; group 3: fibrin gel only, and group 4: untreated injured control. Immediately after alkali injury and peritomy, the cornea surface was extensively rinsed normal saline. The constructs were trimmed to the appropriate size covering the corneal surface and placed with fibrin gel in contact with the stromal bed. The implant was secured with 8 interrupted sutures using 10/0 nylon suture equably on the conjunctiva. Eyelids were closed with 2 temporary tarsorrhaphy sutures using 6/0 silk, which were removed after 7 days. All operated eyes received topical tobramycin (1%, Alcon, Geneve, Switzerland) and dexamethasone phosphate (0.1%, Alcon), 4 times daily. Rats were sacrificed after 4 weeks by overdosed pentobarbital (85 mg/kg; Jurox, Rutherford, NSW, Australia) intraperitoneally and corneas were harvested for immunohistochemistry.

### Ophthalmic examination and measurements

All corneal imaging and measurements were conducted 3 days prior to injury (pre-operative) and weekly post-transplantation for a total of 4 weeks. Slit-lamp micrographs were taken using a Zoom Slit Lamp NS-2D (Righton, Tokyo, Japan). Two independent observers (FB, GY) graded the corneal clarity and haze formation from 0 to 4 under slit-lamp examination [20]. Grade 0 corresponded to a totally clear cornea; grade 1 to minimal haze under direct and diffuse illumination; grade 2 to mild haze easily visible under direct focal slit illumination; grade 3 to moderate haze/opacities that partially obscured the iris details; and grade 4 to severe dense opacities that obscured completely the details of the anterior segment. Corneal epithelial wound closure was assessed by staining with 1% Minims fluorescein sodium solution (Bausch & Lomb, Rochester, NY, USA) for 5 s, followed by sterile saline rinsing, and visualized by digital camera photography in cobalt blue light with ultraviolet light filters [21]. The wound area (with fluorescein label) was determined by a color threshold technique of ImageJ (Fiji) software and expressed as a percentage of total corneal area. Corneal cross-section visualization and measurement of central corneal thickness (CCT) were done using anterior segment optical coherence tomography (AS-OCT, RT-Optovue™, Carl Zeiss Meditec, Dublin, CA, USA). CCT was calculated as the mean of 3 measurements taken at the corneal midpoint and at 0.3 mm on either side [22].

### Rat cornea processing for histochemistry and immunofluorescence

Excised corneas were fixed in neutral-buffered 4% paraformaldehyde for 2 h and cut into equal halves. One part was embedded in Optimum Cutting Temperature (OCT) cryo-compound (Leica Microsystems, Nussloch, Germany) and cryosections (6 μm thick) were obtained for immunofluorescence using antibodies against CDH1, CDH2, CK3, and CK12 (Additional file 1: Table S1). The other corneal half was fixed in 10% formalin (Sigma) for overnight, buffer washed, dehydrated, and processed for paraffin embedding. Sections (5 μm thick) were dewaxed, hydrated, stained with hematoxylin and eosin reagents, and viewed under light microscopy (Axioplan 2).

### Statistical analyses

All data were expressed as mean ± SD. Results from in vivo experiments were analyzed with Prism 6.0 software (GraphPad, San Diego, CA, USA). A comparison of fold changes of marker expression between cell types was assessed by the Mann-Whitney *U* test. Statistical differences for corneal wound areas were determined by ANOVA. *P* < 0.05 was considered statistically significant.

## Results

### Human ADSC characterization

Primary human ADSC were propagated in MesenPRO™ medium to passage 2. Flow cytometric analysis showed high expression rate of standard MSC markers, which were CD73 at 99.5 ± 0.3%, CD90 at 92.2 ± 12.4%, CD105 at 99.4 ± 0.5%, and CD166 at 99.5 ± 0.4%. The expression rate of monocyte-related CD14 was < 4% and hematopoietic-related CD31 was 0.8% (Additional file 1: Figure S1). Multipotency was demonstrated by defined differentiation protocols for adipogenesis, chondrogenesis, and osteogenesis. The treated cells under respective conditions were stained positive to Oil Red O for adipocytes, toluidine blue for chondrocytes, and Alizarin Red S for osteocytes (Additional file 1: Figure S2).

### Mesenchymal-epithelial transition of human ADSC to express epithelial phenotype

**Fig. 2:**
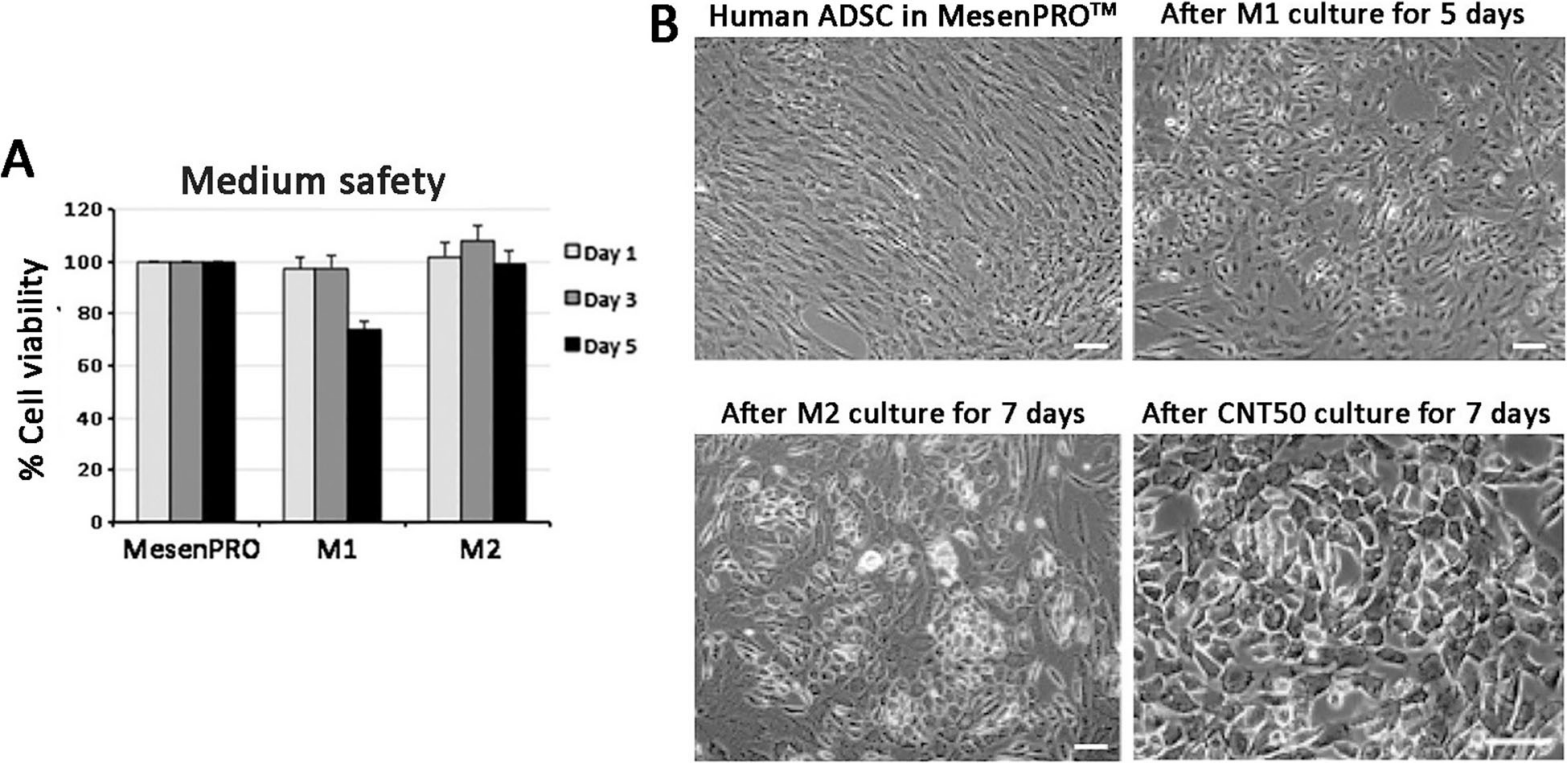
Small molecule treatment of human ADSC to MET-Epi cells. **a** Cell viability measured by MTT assay showed M2 medium caused negligible toxicity, when compared to M1 medium. **b** Phase-contrast micrographs of (i) human ADSC propagated in MesenPRO™ medium, (ii) MET cells after M1 culture for 5 days, (iii) MET cells after M2 culture for 7 days, and (iv) MET-Epi cells after CNT50 culture for 7 days. Scale bars 50 μm

**Fig. 3:**
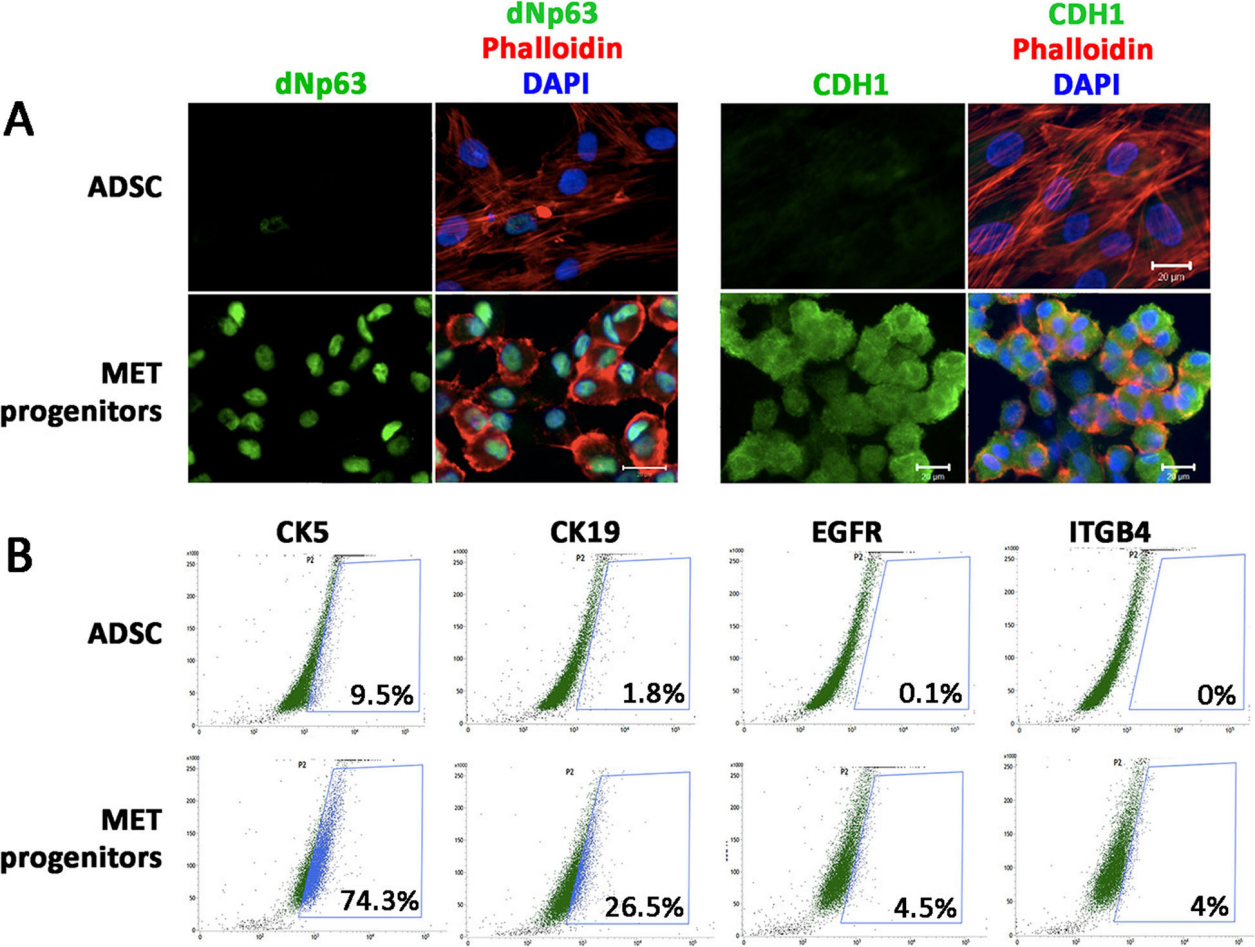
Characterization of MET progenitors. **a** Immunofluorescence showing an induction of nuclear dNp63 and CDH1 (located in the cytoplasm and cell surface) in MET progenitors. Phalloidin was expressed in both human ADSC and MET progenitors. **b** Flow cytometry analysis demonstrating the elevated number of MET progenitors expressing CK5, CK19, EGFR, and ITGB4. Scale bars 10 μm

CD73^high^/CD90^high^/CD105^high^/CD166^high^/CD14^negative^/CD31^negative^ ADSC were treated with small molecules inhibiting GSK3 and TGFβ signaling together w*i*th atRA, as previously reported [19] with minor modifications (Fig. 1). After M1 culture for 5 days, the cells were placed in a revised step with a 7-day culture in M2 medium, which had similar formulation as M1, except the reduced concentration of VPA at 50 μM and CHIR99021 at 300 nM. This adjustment substantially improved cell viability. Using MTT assay, the cell viability after M1 culture step was 73.6% and this was greatly improved when the cells were changed to M2 condition (99.3% viability) (Fig. 2a). Phase-contrast micrographs showed that M2 cultured cells had similar morphology as M1 cultured cells. Both cultures were densely packed and cells were polygonal in shape (Fig. 2b). In contrast, ADSC under MesenPRO™ culture were slender and bipolar in shape with the presence of intercellular space. Under immunofluorescence, M2 cultured MET progenitors expressed CDH1 (E-cadherin, a MET hallmark marker) at both cytoplasmic and cell surface location and δNp63 (a cell proliferation marker of epithelial progenitors) was predominantly nuclear (Fig. 3a). By flow cytometry, there was a general induction of epithelial-related genes after M2 culture (Fig. 3b). CK5 expression was detected in 74% MET progenitors compared to 10% in untreated ADSC. Likewise, CK19 expression was found in 26.5% MET progenitors and only in 1.8% ADSC. Epidermal growth factor receptor (EGFR) was detected in 4.5% MET progenitors but in 0.1% ADSC and integrin-β4 (ITGB4) was expressed in 4% MET progenitors but undetected in ADSC. These results demonstrated that small molecule treatment under M1 and M2 cultures on ADSC generated epithelial-like progenitors through MET.

### Differentiation of MET epithelial progenitors

**Fig. 4:**
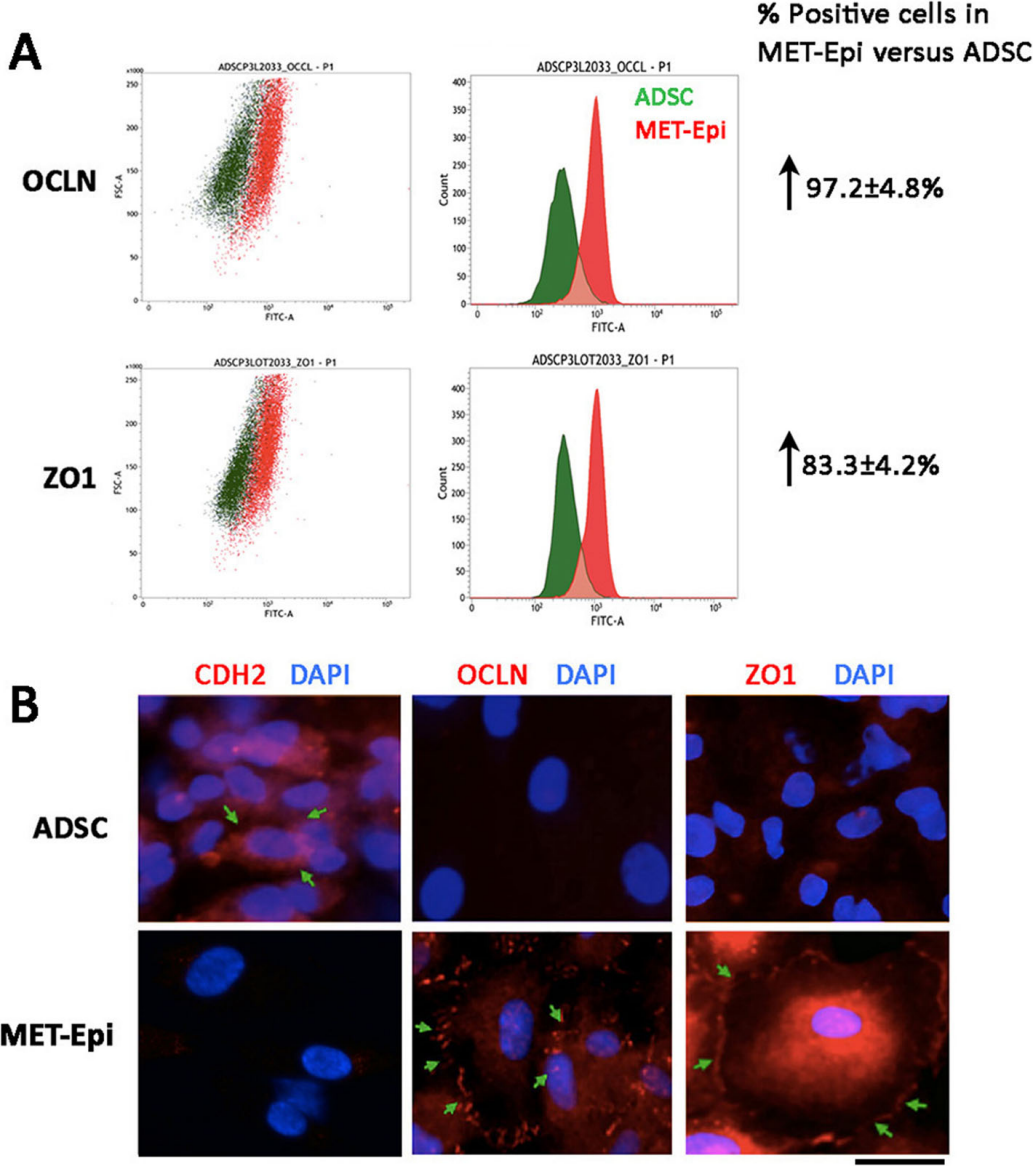
MET-derived epithelial (MET-Epi) cell characterization. **a** Flow cytometry showed an increased number of MET-Epi cells expressing OCLN and ZO1 after CNT50 culture. **b** Immunofluorescence showing the downregulated CDH2 (NCAD) and upregulated OCLN and ZO1 on the cell surface in MET-Epi cells, compared to ADSC. Scale bar 20 μm

The culture of MET progenitors in CNT50 medium gradually induced more epithelial marker expression. Flow cytometric analysis revealed that 97.2 ± 4.8% cells expressed OCLN and 83.3 ± 4.2% cells expressed ZO1 (Fig. 4a). This was also illustrated by immunofluorescence that both OCLN and ZO1 were expressed on the cell surface (Fig. 4b). These cells were termed as MET-Epi cells. In return, the mesenchymal marker, CDH2 (N-cadherin) was downregulated in MET-Epi cells, when compared to ADSC.

### Transplantation of tissue-engineered MET-Epi cells on fibrin gel to alkali injured rat corneal surface

#### Recovery of corneal clarity

**Fig. 5:**
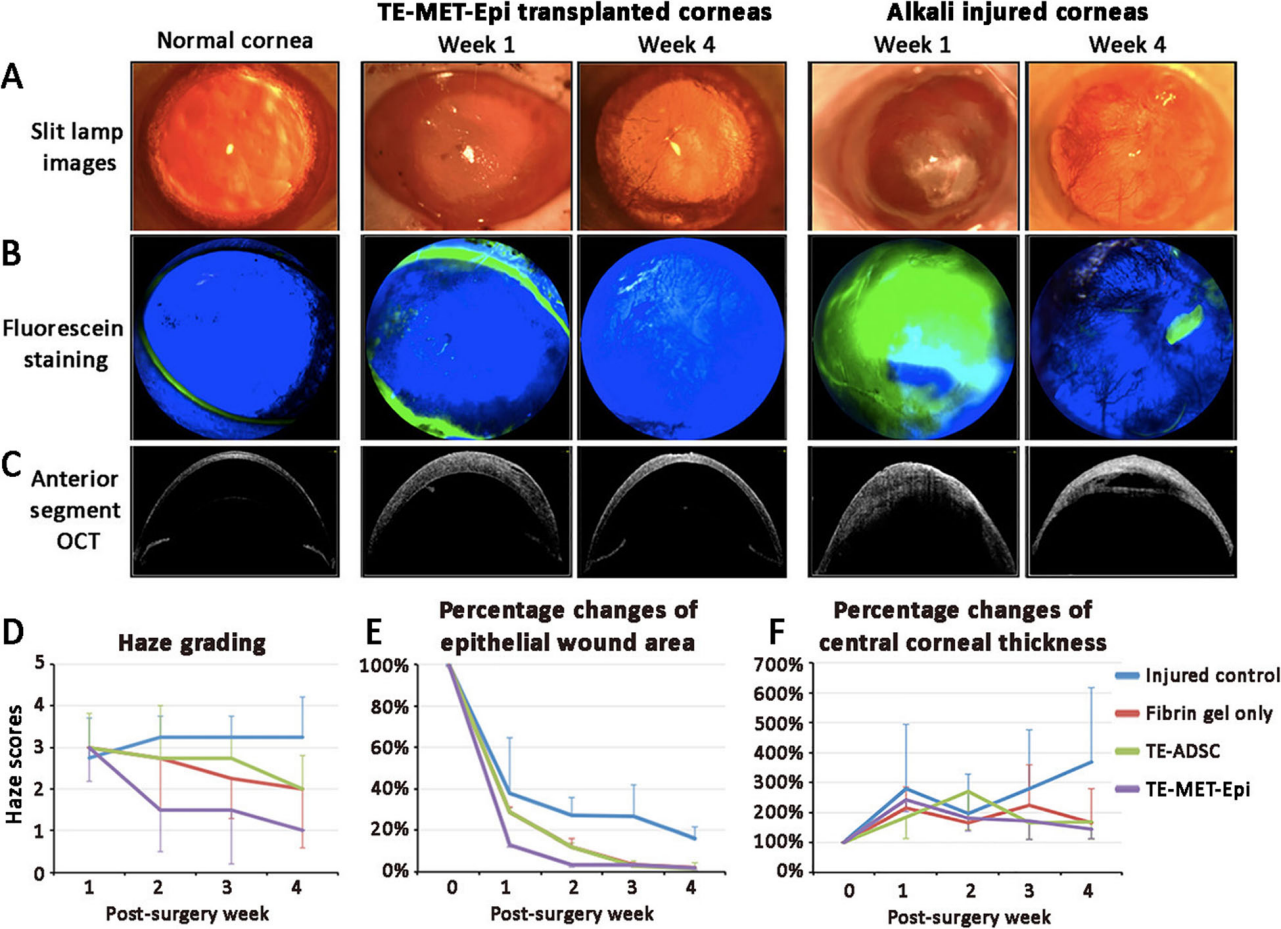
Mouse corneal changes after transplantation of tissue-engineered cell/fibrin gel construct to alkali-injured corneal surface. **a**. Slit-lamp biomicroscopy showing recovery of the clear cornea at week 1 and 4 post-transplantation of TE-MET-Epi constructs. In contrast, alkali-injured corneas were completely opaque and vascularized. **b** Fluorescein staining pictures showing re-epithelialization of corneal surface after TE-MET-Epi transplantation, whereas injured corneas developed epithelial defect and vascularization. **c** Anterior segment OCT showing the corneal thickness after TE-MET-Epi transplantation was similar as the normal cornea and injured corneas remained thicker. **d** Haze grading. **e** Percentage changes of the epithelial defect. **f** Central corneal thickness changes

In the first week post-transplantation, the corneas had considerable haze, which was cleared after 4 weeks (Fig. 5a). The corneas were stable and had minimal neovascularization. Corneal haze grading showed that the corneas at the first-week post-transplantation had a haze score of 3 ± 0.96 (Fig. 5d). This was substantially reduced to score level 1 after 4 weeks (*P* < 0.01, *n* = 6; Mann-Whitney *U* test). In contrast, injured corneas without grafting remained opaque and extensively vascularized. The mean haze scores were above 3 throughout different weeks of examination. Injured rat corneas receiving fibrin gel only and TE-ADSC remained hazy and the scores were greater than 2 up to 4 weeks post-surgery.

#### Improved corneal epithelial healing

Sodium fluorescein stains for corneal stroma but not the intact corneal epithelium, and this demarcates the area with the epithelial loss [23]. When compared to injured corneas without treatment, all groups transplanted with fibrin gel with or without TE cell sheet showed reduced fluorescein intensity, indicating the healing of epithelial defect (Fig. 5b). At 4th week post-surgery, there was 16 ± 5.6% corneal surface with epithelial defects in an untreated injured group whereas < 2% was detected in groups with transplantation (*P* < 0.05) (Fig. 5e). Among different grafts, TE-MET-Epi group showed the fastest epithelial healing. At week 2 post-transplantation, the epithelial defect was 3.2 ± 2.5% for TE-MET-Epi group, whereas TE-ADSC group had 11.8 ± 10.1% and the group with fibrin gel only had 11.8 ± 5.6%. These groups showed similar defect percentages in the third and fourth week (all were < 2%).

#### Restoration of corneal thickness

The cross-section image of corneas under AS-OCT showed that corneal edema occurred in all groups in the first-week post-surgery (Fig. 5c). The mean CCT was increased by 142% in TE-MET-Epi group, 83% in TE-ADSC group, 114% for fibrin gel only and 178% in the untreated injured group (Fig. 5f). The CCT of TE-MET-Epi group gradually decreased to the pre-injury level at the fourth-week post-surgery (*P* < 0.05, compared to untreated injured control). In contrast, corneas receiving TE-ADSC or fibrin gel only had slightly thicker corneas (an increase of 67% for TE-ADSC and 65% for fibrin gel only). The untreated injured corneas remained much thicker (an increase of 269%).

#### Reconstruction of corneal epithelium

**Fig. 6:**
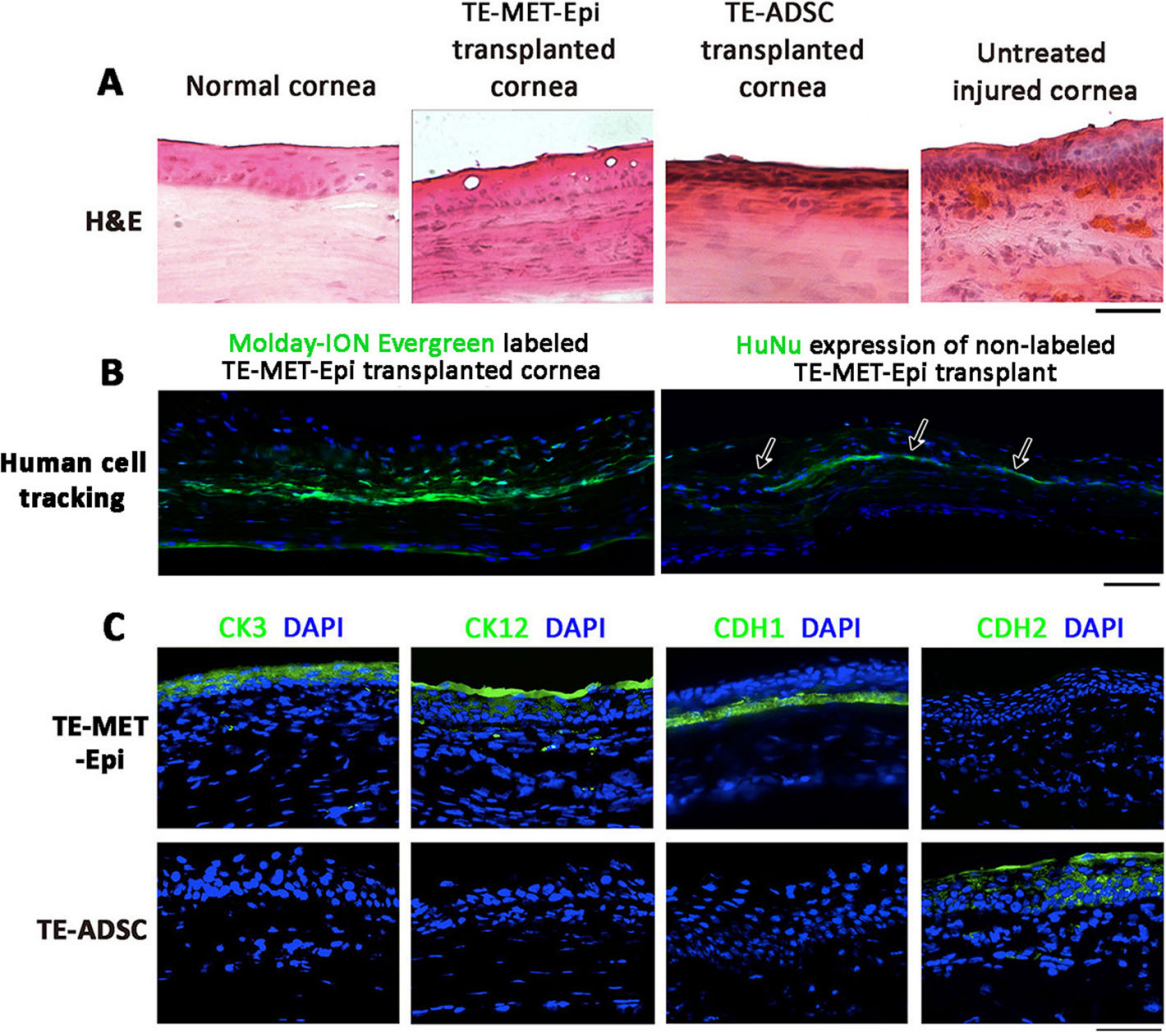
Corneal tissue changes after transplantation of tissue-engineered cell/fibrin gel constructs to the alkali-injured corneal surface. **a**. Representative H&E images of corneas. **b** Homing of Molday-ION Evergreen™ labeled human MET-Epi cells in the cornea (predominantly in basal layers) at week 4 post-transplantation. Similar result was revealed by human-specific HuNu staining (arrows). **c** Immunofluorescence of corneal epithelial marker expression at 4 weeks post-transplantation. CK3, CK12, and CDH1 (E-cadherin) were upregulated in reconstructed epithelial layers after TE-MET-Epi transplantation, but not in TE-ADSC transplanted group. In contrast, CDH2 (N-cadherin) was suppressed in TE-MET-Epi transplanted corneas. Scale bars: 0.1 mm

Histology showed that corneas grafted with TE-MET-Epi for 4 weeks exhibited intact multilayered epithelia, similar to the normal corneas (Fig. 6a). The reconstructed epithelia appeared regular and smooth with 3–5 cell layers in the central region. The detection of Molday-ION Evergreen signals in transplanted rat corneas showed the presence of human MET-Epi cells predominantly in the basal epithelial layers (Fig. 6b). This was validated by a similar expression of human-specific nuclear antigen (HuNu) (Fig. 6b). The keratocytes in the anterior corneal stroma were regularly aligned among the stromal lamellae with minimal detection of immune and inflammatory cells (Fig. 6a). By immunofluorescence, various corneal epithelial markers (CK3, 12, and CDH1) were positively detected, while the expression of CDH2 (mesenchymal marker) was negligible (Fig. 6c). In contrast, rat corneas of TE-ADSC group displayed a thinner cell layer with an irregular thickness (Fig. 6a). There was no expression of CK3, 12, and CDH1, whereas CDH2 was mildly detected (Fig. 6c). In untreated injured corneas, the epithelia were thicker and irregular (Fig. 6a). Polymorphonuclear cells (suggesting lymphocytes) were observed in the basal epithelial and anterior stroma.

## Discussion

In this study, we demonstrated that ADSC could be a viable cell source to generate tissue-engineered (TE) epithelium on bioscaffold for treating persistent epithelial defects (PED) on the corneal surface. The epithelial-like progenitors derived from human ADSC via induced mesenchymal-epithelial transition (MET) were able to differentiate into epithelial cells, which developed into the multilayered epithelium, when cultured on fibrin gel support. The transplantation of this TE-MET-Epi to the corneal surface of a rat total LSCD model, achieved an efficient epithelial healing and suppressed corneal edema and opacities, when compared to injured corneas without treatment or transplanted with TE-ADSC constructs.

The clinical management of bilateral total LSCD is very challenging. Patients often present with PED and corneal instability, e.g., large ulcers that are prone to infection, and have a high risk of corneal melting and perforation [24–26]. Other aggravating conditions, such as lacrimal dysfunction, conjunctivalization, neovascularization, and keratinization, are also common and associated with a higher risk of graft failure in the event of keratoplasty [27]. The criteria of clinical success are the resolution of PED, regression of conjunctivalization and vascularization, and re-establishment of a stable and transparent corneal epithelium (CE). Few treatment options have been shown to be relatively effective for total LSCD.

Allogeneic keratolimbal grafting is a reconstructive surgical procedure but is accompanied by a risk of rejection due to HLA incompatibility, hence it requires prolonged systemic immune suppression [28]. COMET (cultivated oral mucosal epithelium transplantation) using autologous epithelial cells of non-ocular origin is an option to overcome these drawbacks while re-establishing a stable and transparent epithelium [29–32]. However, the corneal surface stability declined gradually in the first 6 months and remained stable in only 50 to 60% of treated eyes thereafter. The failed cases had a recurrence of CE defects and ocular complications, such as corneal melting, perforation, and neovascularization [25, 33]. An alternative option is a Boston keratoprosthesis, however, it is an invasive procedure, which changes the eye anatomy and physiology drastically and irreversibly [34, 35]. Despite the improvements in visual function, there are frequent risks of ocular complications, such as glaucoma development and progression of pre-existing glaucoma [36, 37]. Hence, vigorous peri-operative management and long-term follow-up are required**.** Alternative procedures that provide satisfactory visual rehabilitation with less complications and long-term corneal stability are needed.

Renewal cell sources of embryonic stem (ES) cells and induced pluripotent stem cells (iPSC) have been explored for stem cell-based therapy in wounded corneas [38]. Several reports have illustrated the differentiation potency of these pluripotent cells to CE-like cells via ex vivo induction with cytokines and growth factors, as well as extracellular support [39–42]. Nonetheless, the critical issues concerning the risk of tumorigenesis, high procedure cost, and the reproducibility and homogeneity of epithelial differentiation remain to be tackled before any consideration for clinical use [43, 44]. Other studies using hair follicle stem cells, dental pulp stem cells, and umbilical cord stem cells are still in the experimental stage and further investigation is required to refine and improve the differentiation efficiency towards CE lineage [45–47].

MSC represent a non-immunogenic source of multipotent stem cells that are available in adult tissues. Several preclinical and clinical studies have reported their potential therapeutic values in healing and regenerating CE, improving corneal transparency and vision [48–50]. Human ADSC were well-tolerated on rabbit corneas, and they reduced corneal inflammation, neovascularization, and opacity in a rabbit LSCD model [51]. Moreover, epithelial-like cells transdifferentiated from rabbit ADSC using limbal condition media culture regenerated clear and smooth corneas with epithelial-specific CK3/12 expression in rabbit corneas having LSCD [52]. In a clinical trial of 28 LSCD patients receiving allogeneic bone marrow MSC and allogeneic cultivated limbal epithelium, respectively, MSC transplantation was safe and improved the CE phenotype in 71% cases, which was comparable to cultivated limbal epithelium (CE reformation in 67% cases), at 12 months post-surgery (ClinicalTrials.gov number, NCT01562002) [16]. These results favor the application of MSC in healing the diseased CE due to LSCD.

The underlying effects of MSC on the damaged ocular surface can involve multiple mechanisms [9]. Whether MSC can transdifferentiate into CE cells is still uncertain. On the other hand, the capacity of MSC in secreting trophic and growth factors, small RNAs via extracellular vesicles or exosomes, could exert anti-inflammatory and immunomodulatory effects and to stimulate resident viable cells to survive and proliferate, hence reducing tissue injury [10, 17]. In addition, the secretome of ADSC can suppress epithelial-mesenchymal transition (EMT) of diseased CE, in attenuating fibrosis and restoring the corneal transparency [53].

Our work specifically focused on MSC transdifferentiation into CE cells. Previously, we reported an ex vivo culture of human ADSC with a combination of small molecules antagonizing GSK3 and TGFβ signaling to generate epithelial-like progenitors with substantially upregulated CDH1 (E-cadherin) and downregulated mesenchymal gene CDH2 (N-cadherin) expression [19], which is the hallmark of MET [54]. In the present study, these MET progenitors were proliferative to generate cells expressing δNp63 (an epithelial proliferation marker), CDH1, CK5 and 19, epidermal growth factor receptor (EGFR), and integrin-β4 (ITGB4), indicating a commitment to epithelial lineage. When cultured under a defined progenitor cell-targeted (PCT)-epithelial differentiation condition, these MET progenitors formed intact epithelium with densely populated cobblestone-like cells expressing cell junction proteins, ZO1, and occludin. Hence, human ADSC, via an intermediate MET progenitor stage, were able to proliferate and differentiate into cells expressing epithelial-associated phenotype and further generate confluent epithelia.

To prepare for translational use on the corneal surface, these MET progenitors were able to adhere and proliferate on a cross-linked fibrin gel, which acts as a biological substrate to support cell expansion and differentiation [55]. To maintain the intact epithelium on fibrin gel, we added a protease inhibitor aprotinin to prevent gel degradation during culture [56]. This fabrication of tissue-engineered (TE) epithelium from MET progenitors created a transportable, pliable, and stable construct (TE-MET-Epi) ready for transplantation. There was competing interest in this invention.

Using a rat alkali-induced total LSCD model, the transplantation of TE-MET-Epi was shown to restore CE and stabilize the ocular surface. Up to 4 weeks post-surgery, human MET-Epi cells prelabeled with Molday-ION Evergreen™ were detected and predominantly homed to the basal epithelial layers, indicating the role in maintaining the multilayered corneal epithelium. This could associate with the faster epithelial healing and better resolution of corneal edema and haze in the TE-MET-Epi group than others with TE-ADSC, fibrin gel only, or untreated control. The central corneal thickness and haze score were close to the pre-operative values. On the treated corneas of TE-MET-Epi group, the cells colonizing on the corneal surface expressed CDH1 (ECAD) but not CDH2 (NCAD), which indicated the epithelial feature; and this was consistent to our previous results for MET progenitors [19]. The generated epithelia also expressed standard CE markers (CK3 and 12) that were undetectable for rat conjunctival cells. In contrast, rat corneas in TE-ADSC group expressed CDH2 but not CK3 and 12, indicating that they were unable to generate specific CE phenotype. For the alkali-injured cornea controls, the expression of CK4 and 19 illustrated the conjunctival epithelial cells, as a result of conjunctivalization, a consequence of conjunctival epithelial invasion after limbal destruction. In conclusion, the epithelial-like progenitors derived from ADSC, via MET, could differentiate into cells expressing CE markers and form an intact CE in restoring epithelial defects of LSCD.

In our model, the treatment outcome could be influenced by 1 week of tarsorrhaphy (eyelid closure); however, this was essential to prevent implant dislocation, as the rats might rub their eyes vigorously; and the same procedure was performed in all groups. The closed corneal surface reduced the exposure of MET-Epi cells to air-liquid interphase that could have hindered the epithelial cell polarization and differentiation [57]. Further evaluation using large animals may avoid the procedure of eyelid closure and the implant could be covered and protected by amniotic membrane [58]. In addition, ADSC are known to have the potential to augment angiogenesis [59], which functionally recovers ischemia-induced injuries [60]. However, this effect seemed to be diminished in MET-Epi cells derived from ADSC, and the transplanted corneas had reduced corneal neovascularization. Our further work will investigate the effect of MET on the expression of paracrine factors from ADSC on pro-angiogenesis (e.g., vascular endothelial growth factor-A, platelet-derived growth factor BB, basic fibroblast growth factor) and anti-angiogenesis (e.g., thrombospondin-1), and how these changes influence the corneal epithelial regeneration and stability. For potential clinical application, the TE epithelia from ADSC-derived MET cells can be fabricated in an autologous and xenofree fashion. Bioengineered recombinant human fibrin is safe without the risk of blood-borne pathogen transmission, has equivalent or better tensile strength than plasma-derived fibrin, and can be amenable to large-scale production [61]. It has been shown to maintain MSC viability and growth behavior, and cell delivery to the target site [62]. Our study showed that ADSC-derived epithelial cells on fibrin gel construct were able to recover the defective corneal surface. Further studies will involve the long-term and quantitative assessment of CE recovery and stability as well as corneal haze reduction.

## Conclusion

In this study, we demonstrated that human ADSC-derived MET progenitors generated TE-MET-Epi constructs, which significantly improved the CE recovery with efficient re-epithelialization and a more stable corneal surface, compared to other treatment arms, in a rat alkali-induced LSCD model. These results add to the field of stem cell therapy for corneal surface disorders by confirming the safety, non-immunogenicity, and potential clinical advantages using this mode of stem cell-based treatment for corneal epithelial disorders associated with bilateral LSCD.

## Supplementary information

**Additional file 1:** **Figure S1.** Human ADSC characterization. **Figure S2.** Human ADSC characterization using trilineage cell differentiation. **Table S1.** Primary antibody information.

## Data Availability

All data are included in the text and supplementary information.

## Abbreviations

ADSC: Adipose mesenchymal stem cells
ASOCT: Anterior segment optical coherence tomography
BSA: Bovine serum albumin
CCT: Central corneal thickness
CE: Corneal epithelium
CK: Cytokeratin
EGFR: Epidermal growth factor receptor
EMT: Epithelial-mesenchymal transition
GSK3: Glycogen synthase kinase 3
ITGB4: Integrin β4
LSCD: Limbal stem cell deficiency
MET: Mesenchymal-epithelial transition
NGS: Normal goat serum
MSC: Mesenchymal stem cells
PED: Persistent epithelial defect
TE: Tissue-engineered
TGFβ: Transforming growth factor β
