# Supplementary material for: Cellular therapy of corneal epithelial defect by adipose mesenchymal stem cell-derived epithelial progenitors
Source: Stem Cell Res Ther. 2020 Jan 3;11:14. doi: 10.1186/s13287-019-1533-1 (PMC6942321; doi:10.1186/s13287-019-1533-1)
Supplement: Supplementary file 1 — Additional file 1: Figure S1. Human ADSC characterization. Figure S2. Human ADSC characterization using trilineage cell differentiation. Table S1. Primary antibody information. [file 13287_2019_1533_MOESM1_ESM.docx]

**Cellular therapy of corneal epithelial defect by adipose mesenchymal stem cell-derived epithelial progenitors**

Francisco Bandeira, Tze-Wei Goh, Melina Setiawan, Gary Hin-Fai Yam, Jodhbir S Mehta

**Additional file 1**


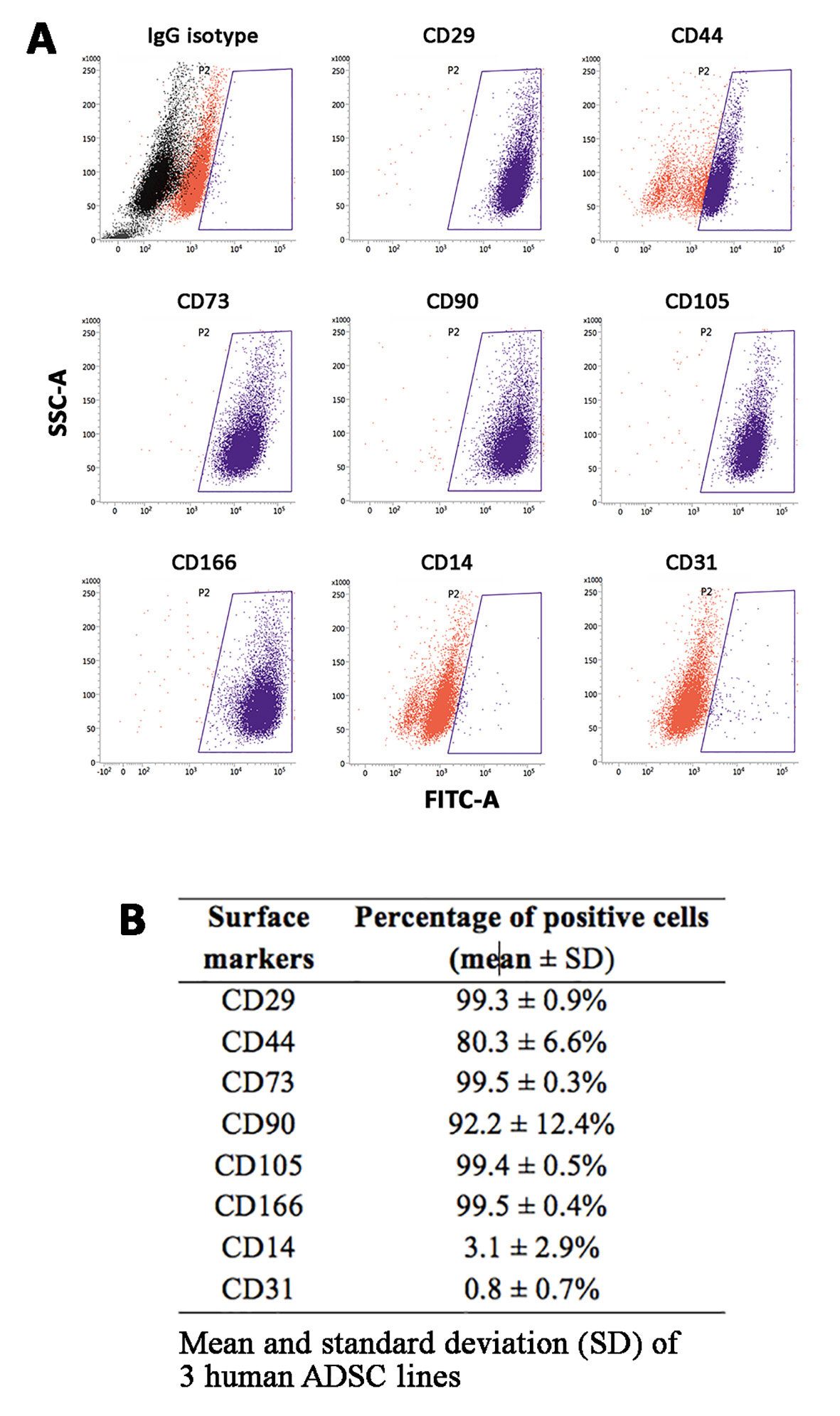


**Figure S1. Human ADSC characterization. A**. Flow cytometry analysis using scattered plots showing the expression profiles of various cell surface markers. **B**. Percentages of cells expressing CD markers.


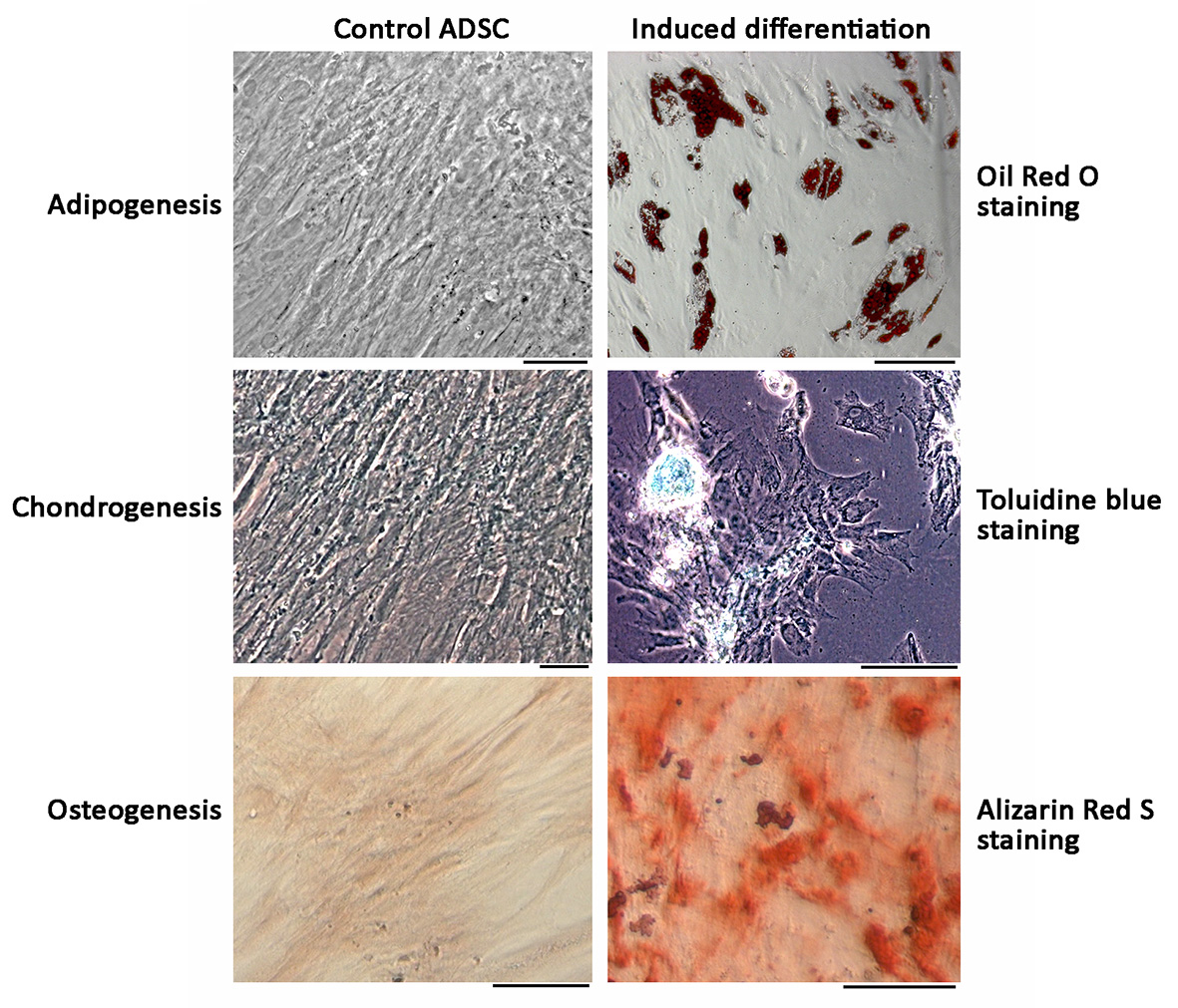


**Figure S2. Human ADSC characterization using trilineage cell differentiation. A.** Adipogenesis and Oil red O staining. **B.** Chondrogenesis and toluidine blue staining. **C.** Osteogenesis and Alizarin Red S staining. Scale bars: 150 μm.

**Table S1. Primary antibody information**

| **Antibody [clone]** | **Isotype** | **Company**  **(catalogue no.)** | **Applications** |
| --- | --- | --- | --- |
| CD14 [#134620] | Mouse IgG_1_ | R&D (MAB3832) | FC: 0.2 μg/10^6^ cells |
| CD29 [18] | Mouse IgG_1_ | BD (610468) | FC: 0.2 μg/10^6^ cells |
| CD31 [WM59] | Mouse IgG_1_ | BD (555444) | FC: 0.2 μg/10^6^ cells |
| CD44 [BF24] | Mouse IgG_1_ | Abcam (ab27284) | FC: 0.2 μg/10^6^ cells |
| CD73 [4G4] | Mouse IgG_1_ | Novus (NBP1-60135) | FC: 0.2 μg/10^6^ cells |
| CD90 [5E10] | Mouse IgG_1_ | BD (550402) | FC: 0.2 μg/10^6^ cells |
| CD105 [266] | Mouse IgG_1_ | BD (555690) | FC: 0.2 μg/10^6^ cells |
| CD166/ALCAM | Mouse IgG_1_ | BD (105902) | FC: 0.2 μg/10^6^ cells |
| CDH1/E-cadherin [67A4] | Rabbit IgG | Sigma (SAB4700237) | FC: 0.2 μg/10^6^ cells IF: 0.5 μg/ml |
| CDH2/N-cadherin [GC-4] | Mouse IgG_1_ | Sigma (C2542) | FC: 0.2 μg/10^6^ cells IF: 0.5 μg/ml |
| Cytokeratin 3/CK3 [AE5] | Mouse IgG_1_ | Abcam (ab77869) | IF: 0.5 μg/ml |
| CK5 [2C2] | Mouse IgG_1_ | Abcam (ab128190) | IF: 0.5 μg/ml |
| CK12 [EPR1609] | Rabbit IgG | Abcam (ab124975) | IF: 0.5 μg/ml |
| CK19 [RCK108] | Mouse IgG_1_ | Millipore (MAB3238) | IF: 0.5 μg/ml |
| Epidermal growth factor receptor/ EGFR [7F12.1] | Mouse IgG_2b_ | Sigma (MABS835) | FC: 0.2 μg/10^6^ cells |
| Human specific nuclear antigen (HuNu) [235-1] | Mouse IgG_1_ | Millipore (MAB1281) | IF: 0.2 μg/ml |
| Integrin β4/ITGB4 | Mouse IgG_1_ | Sigma (MAB1964) | FC: 0.2 μg/10^6^ cells |
| Occludin [19] | Mouse IgG_1_ | BD (611091) | FC: 0.2 μg/10^6^ cells IF: 0.5 μg/ml |
| dNp63 | Rabbit IgG | Biolegend (619002) | IF: 0.5 μg/ml |
| ZO1 | Rabbit IgG | Millipore (AB1554) | FC: 0.2 μg/10^6^ cells  IF: 2 μg/ml |
| Mouse IgG_1_ isotype control |  | SantaCruz (sc-3877) | IF: 1:500 |
| Mouse IgG_2_ isotype control |  | SantaCruz (sc-3878) | IF: 1:500 |
| Rabbit IgG isotype control |  | Invitrogen (08-6199) | IF: 1:500 |

* IF: immunofluorescence; Im-SEM: immuno-scanning EM; WB: western blotting
